# Fatal Coronary Artery Anomaly Concealed in Young Athletes with Exertional Syncope

**DOI:** 10.1155/2024/6390066

**Published:** 2024-02-01

**Authors:** Toshinobu Ifuku, Keigo Nakatani, Kentaro Ueno, Naoto Yamashita, Yutaka Imoto

**Affiliations:** ^1^Department of Pediatrics, Miyazaki Prefectural Miyazaki Hospital, Miyazaki, Japan; ^2^Department of Pediatrics, Kagoshima University Graduate School of Medical and Dental Sciences, Kagoshima, Japan; ^3^Department of Cardiovascular and Gastroenterological Surgery, Kagoshima University Graduate School of Medical and Dental Sciences, Kagoshima, Japan

## Abstract

**Background:**

Syncope is a common symptom in children, many of which are benign and do not require treatment. Anomalous aortic origin of a coronary artery (AAOCA) is a rare congenital malformation but can be a risk for serious cardiovascular events, including sudden death as well as cardiogenic syncope. *Case Report*. We describe the case of a 14-year-old boy who suffered an initial syncope and afebrile seizure during a soccer game. A detailed medical history and imaging studies led to the diagnosis of the anomalous aortic origin of the left main coronary artery with an intramural course (AAOLCA-IM).

**Conclusion:**

Symptomatic AAOLCA-IM has the highest risk of sudden death among AAOCA, and surgical repair may be performed. Onset during exercise or preceding chest symptoms are suspicious signs of cardiogenic syncope and should be considered for cardiovascular imaging evaluation.

## 1. Introduction

Syncope is a common complaint in children. Many of its causes are benign and do not require treatment. However, cardiogenic syncope can lead to serious conditions and even sudden death. Anomalous aortic origin of a coronary artery (AAOCA) occurs in the frequency of 0.3–1% and varies from asymptomatic to life-threatening complications, depending on the morphology [1]. Anomalous aortic origin of the left main coronary artery with an intramural course (AAOLCA-IM) is a rare malformation and carries a high risk of myocardial ischemia and sudden death. Hence, cardiac surgery may be recommended in such cases, but the indications for treatment remain controversial [2–5]. We report a case of AAOLCA-IM diagnosed after initial syncope during exercise and highlight the importance of medical history and confirming the diagnosis at the onset of symptoms.

## 2. Case Report

A 14-year-old boy was referred to our hospital for a detailed examination of initial syncope and afebrile seizures. Three days prior to the presentation, the patient suddenly collapsed during a soccer game and developed generalized tonic seizures with median ocular fixation. The seizures spontaneously ended after approximately 10 minutes, during which no effective cardiopulmonary resuscitation by the bystander was required. An ambulance arrived soon thereafter, and he was nearly alert. Vital signs were stable, and he was transported to a nearby hospital. Physical examination revealed no trauma, dehydration, or respiratory, circulatory, or neurological abnormalities. Electrocardiogram showed no arrhythmias or ST-T abnormalities. Head magnetic resonance imaging and general blood tests, including cardiac enzyme measurements, were unremarkable. He was hospitalized for observation and discharged the following day. The hospital was unable to perform a thorough cardiovascular examination and was further away from his home, so he was referred to our facility. His past medical history included mild asthma and no obvious cardiac disease, arrhythmia, or family history of sudden death.

**Figure 1:**
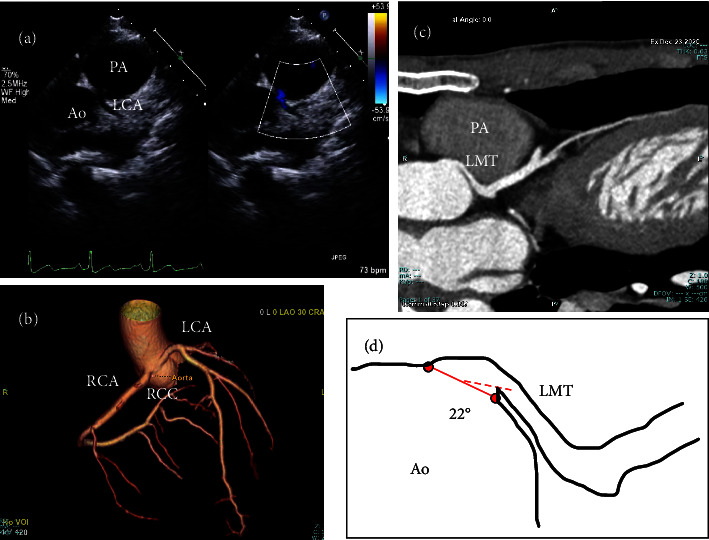
(a) Transthoracic echocardiography (parasternal short axis) at initial examination. The origin of pain in the left coronary artery is not detected in its normal position. (b) Coronary computed tomography angiography (CCTA, three-dimensional reconstruction) depicting the left coronary artery originating from the right sinus of Valsalva. (c) CCTA shows that part of the left main coronary artery trunk runs between the aorta and pulmonary artery. (d) Measurements of the take-off angle from the ascending aorta in the left coronary artery. The solid line indicates the so-called ostium line, and the dashed line indicates the line drawn from the midpoint of the ostium line to a point along the center of the left main trunk. The angle between the two lines at the midpoint is measured as the take-off angle and is 22 degrees. Ao: aorta; PA: pulmonary artery; LCA: left coronary artery; RCA: right coronary artery; RCC: right coronary cusp; Left main coronary trunk.

Medical interviews indicated that during a soccer game, the patient experienced gripping pain in his anterior chest, followed by a blackout but was conscious just before the collapse. In addition, he had recently experienced brief chest discomfort several times during exercise. Based on this information, we suspected cardiogenic syncope and performed echocardiography, which failed to identify the origin of pain in the left coronary artery in its normal position (Figure 1(a)). Coronary computed tomography angiography (CCTA) demonstrated that the origin of the left coronary artery was located in the right sinus of Valsalva, and part of the left main trunk was intramural (Figures 1(b) and 1(c)). The left coronary artery had a very acute take-off angle from the aorta and the ostium was slit-like (Figure 1(d)). Electroencephalography was normal. Both the treadmill exercise test (Bruce protocol) and myocardial scintigraphy (resting and exercise stress) reached the target heart rate of 175 bpm but did not induce chest symptoms, myocardial ischemia, or arrhythmia.

**Figure 2:**
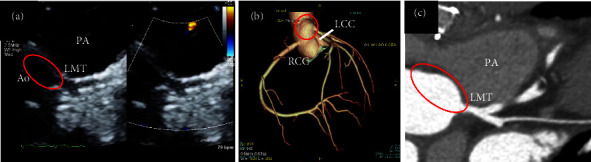
Transthoracic echocardiography (a) and computed tomography angiography (b, c) 6 months after surgery showing the new left coronary ostium with unroofing of the intramural portion and patch enlargement using the great saphenous vein graft (red circles). Ao: aorta; PA: pulmonary artery; LMT: left main coronary artery trunk; LCC: left coronary cusp; RCC: right coronary cusp.

AAOLCA-IM was diagnosed and the patient was referred to a specialized facility for early cardiac surgery. He and his parents agreed to this policy due to a malignant coronary artery anomaly and to continue playing soccer. Exercise was prohibited until surgery. The surgical procedure was as follows. The intramural segment was unroofed by excising the common wall until the coronary artery exited the aorta from the left sinus of Valsalva. The new left coronary artery orifice was slightly narrowed and subsequently augmented via patch enlargement by using a great saphenous vein graft (Figure 2). The postoperative healing was uneventful, and the patient was discharged 2 weeks later. Aspirin was administered as an antiplatelet therapy for 6 months, and exercise restrictions gradually eased. He underwent regular outpatient checkups and was able to play soccer without any symptoms.

## 3. Discussion

Vasovagal syncope and postural orthostatic tachycardia syndrome account for approximately 75% of syncope cases in children, whereas cardiogenic syncope accounts for approximately 10%. It is estimated that 5% of patients with syncope have convulsive seizures [6]. In autopsy cases of young competitive athletes, AAOCA was reported to be the second most common cause of sudden cardiac death [7]. AAOCA has accumulated data primarily from symptomatic cases and autopsies, but recent advances in imaging techniques have led to the reporting of many asymptomatic cases. Controversy remains regarding its therapeutic management and long-term prognosis.

The exact mechanism by which AAOCA causes ischemia and syncope remains unknown. Conventionally, myocardial ischemia was thought to be induced by hemodynamic changes in addition to fixed stenosis of the coronary arteries, and strenuous exercise and acute take-off angle ≤30° in adolescents were considered high risk. Some adult cases have been reported to be associated with coronary spasm [1]. However, even forms that have been considered high risk may pass asymptomatically or occasionally and do not induce ischemic symptoms in exercise or pharmacologic stress tests [3].

Recent reports have proposed a mechanism in which global ischemia of the left ventricle causes a decrease in cardiac output with bradycardia or cardiac arrest, leading to physical collapse [8]. Our patient did not require resuscitation, including defibrillation. Myocardial ischemia or ventricular tachyarrhythmia improved in a short time, which may explain the absence of abnormal electrocardiogram and blood tests after the syncope. He was aware of chest symptoms during exercise and may have experienced several short-term episodes of myocardial ischemia.

Cardiac surgery is not mandatory in symptomatic AAOLCA, but there is a consensus that it may be considered. Moreover, the determination of the indication for treatment is increasingly weighted on whether the presence of myocardial ischemia can be demonstrated [4, 5]. The present case opted for surgical treatment due to a history suggesting recurrent myocardial ischemia and a desire to continue to play soccer, which he is passionate about.

Syncope during or immediately after exercise is rare (3% incidence in children and adolescents) [9], and the differential diagnosis should include cardiogenic syncope involving hereditary arrhythmias, cardiomyopathies, or cardiovascular malformations. A detailed medical history can provide clues to differentiate the causes of syncope. Echocardiography and CCTA are also useful for evaluating the coronary artery morphology.

## 4. Conclusion

Symptomatic AAOLCA-IM has the highest risk of sudden death among AAOCA and requires consideration for surgical treatment. Cardiogenic syncope in children is infrequent but carries a risk of serious cardiovascular events, including sudden death. Onset during exercise or preceding chest symptoms are suspicious signs of cardiogenic syncope and should be considered for cardiovascular imaging evaluation.

## Data Availability

No data were used to support the findings of this study.
